# Welfare Assessment of Female BALB/c Mice Housed with Different Environmental Enrichment Materials

**DOI:** 10.3390/ani16142211

**Published:** 2026-07-16

**Authors:** Rosa De Jesús, Patricia L. Fernández, Cesar Murillo, Juan De Dios Noriega, Alanna Madrid, María B. Carreira

**Affiliations:** 1 Unidad de Experimentación Animal, Instituto de Investigaciones Científicas y Servicios de Alta Tecnología (INDICASAT AIP), Ciudad del Saber, Panama City 7144, Panama; amadrid@indicasat.org.pa; 2 Sistema Nacional de Investigación (SNI AIP), Ciudad del Saber, Panama City 7144, Panama; pllanes@indicasat.org.pa; 3 División de Salud y Enfermedades Humanas, Instituto de Investigaciones Científicas y Servicios de Alta Tecnología (INDICASAT AIP), Ciudad del Saber, Panama City 7144, Panama; 4 Escuela de Biología, Facultad de Ciencias Naturales, Exactas y Tecnología, Universidad de Panamá, Panama City 7156, Panama; cesarmurillonavas@gmail.com; 5 Escuela de Biotecnología, Facultad de Ciencias de la Salud Dr. William C. Gorgas, Universidad Latina de Panamá, Panama City 7155, Panama; noriegajuandedios28@gmail.com

**Keywords:** enrichment materials, BALB/c mice, animals’ welfare, animal facilities, environmental enrichment

## Abstract

Environmental enrichment is widely recognized as an important strategy for improving the welfare of laboratory rodents by promoting species-specific behaviors, reducing stress, and enhancing physiological adaptation. In the present study, animals were maintained either under standard housing conditions without enrichment or exposed to different combinations of commercially available maple cardboard, cardboard rolls, and Kraft paper. Welfare was assessed using a multidimensional approach that included anxiety-related behavioral tests, hematological and biochemical analyses, and growth performance measurements. The results demonstrate that the welfare benefits of environmental enrichment are influenced by the type and combination of materials provided, highlighting the importance of selecting enrichment strategies that are appropriate for the specific housing conditions.

## Simple Summary

Environmental enrichment is an important factor in the welfare of laboratory mice, as the type and combination of materials can influence their behavior and physiology. In this study, we evaluated the effects of different enrichment materials—maple cardboard, cardboard rolls, and Kraft paper—on female BALB/c mice housed under standardized conditions. Overall, most enrichment strategies did not produce major changes in welfare indicators. However, the combination of cardboard rolls and maple cardboard appeared to induce mild stress responses, while the use of multiple diverse materials or cardboard rolls plus Kraft paper was associated with reduced anxiety-like behavior and stable physiological outcomes. These findings suggest that enrichment programs should carefully consider the choice and combination of materials to optimize animal welfare.

## 1. Introduction

The main objective of environmental enrichment (EE) is to improve animal welfare, providing sensory and motor stimulation to animals through structures and resources that facilitate the expression of species-typical behaviors. Furthermore, it promotes psychological well-being through physical exercise, manipulative activities, and cognitive challenges according to the specific characteristics of the species [1,2,3]. EE provides animals with the opportunity to have a degree of control over their environment, allowing them to better cope with stressful situations caused by environmental or social factors or by the experimental procedures they undergo [4,5].

Studies have evaluated whether specific environments benefit laboratory mice. Lee et al. [6] report that animals exposed to EE showed less stress compared to those kept without EE, regardless of whether they were housed individually or in groups. This stress reduction was evidenced by a greater body weight gain and lower concentrations of circulating corticosteroids. Duarte et al. [7] describe how the mice used the supplied material as enrichment, making it part of their environment and using it to build their nests, improving the number of offspring per litter, weight gain among offspring, and nest building scores.

However, studies have demonstrated that responses to enrichment vary according to the strain and sex of mice and rats [8,9], while different enrichment strategies can differentially influence behavioral outcomes and hematological parameters [10]. Moreover, these parameters can be affected by the cage type, stocking density, cage size, and macro-environmental conditions [11]. Therefore, the implementation of environmental enrichment should not rely on standardized approaches alone but rather on programs specifically adapted to the characteristics of each animal facility. Establishing facility-specific enrichment programs is essential not only to maximize animal welfare but also to minimize unintended effects on physiological and experimental parameters, directly impacting the reproducibility of research findings and the reduction in the number of experimental animals.

This study evaluated the effect on the welfare of female BALB/c mice of using maple cardboard, cardboard rolls, and Kraft paper as environmental enrichment materials, placed individually or in combination. To understand this effect, we examined growth, behavior in the Elevated Plus Maze and in the open field [12,13], and hematological and biochemical parameters (GLU and ALT) [14,15]. The relevance of this study lies in determining the benefits of using these materials within an institutional environmental enrichment program.

## 2. Materials and Methods

### 2.1. Animals and Housing

This study was designed and conducted following the ARRIVE guidelines. Eighty female BALB/cAnNCrL (21 days old; mean body weight 11.32 g) mice were used. All animals were bred and maintained at the INDICASAT AIP animal facility since 2018 (current generation F18), originally obtained from the Charles Rivers Laboratory (Kingstone, NY, USA).

Mice were housed in individually ventilated cages (IVCs) (Tecniplast, Buguggiate, Italy, model 1285L; floor area: 542 cm^2^), with five animals per cage, in accordance with the Guide for the Care and Use of Laboratory Animals. Room temperature and relative humidity were maintained at 18 ± 1 °C and 66 ± 10%, respectively. Temperature and humidity inside the cages were maintained at 19 ± 1 °C and 66 ± 10%, respectively (Tecniplast ventilation system). A 12/12 h light/dark cycle was used, with a light intensity ranging from 110 to 130 lux.

Animals had ad libitum access to an irradiated pelleted diet (LabDiet 5053, Dayton, OH, USA), and reverse osmosis-filtered water was provided via drinking bottles. Sterilized bedding consisted of 125 g of Bed-o’Cobs ¼” (The Andersons, Galena Park, TX, USA).

### 2.2. Experimental Design

Eight (8) treatment groups were formed at weaning and randomly housed in groups of 5 animals per cages, including 2 cages per treatment for a total of 80 animals (*n* = 10 per treatment group). The animals were housed with different environmental enrichment materials or in standard housing conditions. The enrichment materials consisted of low-cost [5,16,17] recyclable items that were sterilized by autoclaving before use. The experimental groups were as follows: T1—maple cardboard (egg carton compartments) plus cardboard rolls (paper towel or toilet paper tubes) plus Kraft paper, T2—only maple cardboard, T3—only cardboard rolls, T4—only Kraft paper, T5—cardboard rolls plus Kraft paper, T6—maple cardboard plus Kraft paper, T7—cardboard rolls plus maple cardboard, and T8—without environmental enrichment. For the enrichment materials, approximately 20 ± 2 g was placed in each cage. New enrichment material, as appropriate for each experimental group, was provided once a week when the animals were moved to clean cages.

Body weight was recorded weekly for 6 weeks using a precision scale (KERN KB, Balingen, Baden-Württemberg, Germany) and was always recorded between 8:30 and 10:00 a.m. The exposure period was defined as part of the animal welfare program to evaluate the effects of environmental enrichment introduced during early life. In week 7, three days after the final weight measurement, five animals from a randomly selected cage per group (total *n* = 40) were tested in the Open Field (OF), followed by the Elevated Plus Maze (EPM) on the next day. Blood samples were taken from the 80 animals 2–3 days after completion of the behavioral assays for evaluation of hematological parameters (complete blood cell count and biochemical analyses).

### 2.3. Behavioral Evaluation

The trials were conducted between 9:00 a.m. and 12:00 p.m. Prior to the behavioral tests, the animals were housed for 30 min in the conditions of the behavioral testing room, with the aim of allowing them to adapt to the conditions of the room.

Open Field (OF): Mice were exposed to the open field maze for 5 min under red light illumination. The maze consisted of a white plexiglass box measuring 50 cm × 50 cm × 30 cm. For the test, each mouse was placed in the open field border region of the apparatus and allowed to explore freely for 5 min; the behavior was recorded via camera. Behavior was evaluated according to time in the central and peripheral zones and average speed.

Elevated Plus Maze (EPM): The test involved placing each mouse in the center of the cross-shaped maze raised 72.4 cm off the ground, with two opposite open arms (50.8 cm × 10.2 cm) and two opposite closed arms (50.8 cm × 10.2 cm × 40.6 cm) (ENV560; Med Associates, Inc., Fairfax, VT, USA), allowing the mouse to explore freely for 5 min. The following parameters were used to evaluate behavior: time in open and closed arms, entries into open and closed arms, and total entries (locomotor activity). The time that mice spent in the open arms was used as a behavioral parameter to assess anxiety-like behaviors. This behavior was recorded throughout the time period using a camera mounted at the top of the maze. Anxiety-related behavior is associated with less exploration of the open arm in relation to general exploration of all arms.

All data was recorded automatically using the Any-maze video tracking system, version 7.45 (Stoelting Co., Wood Dale, IL, USA).

### 2.4. Hematological and Biochemical Evaluation

Whole blood (~400 µL) was collected by intracardiac puncture under isoflurane (5%) anesthesia, with the thoracic cavity closed. The following aliquots were obtained: 50 µL (EDTA) for hematological analysis and 200 µL (heparin) for biochemical analysis. A sample loss rate of 20–30% due to insufficient blood volume was anticipated during blood collection. Hematological analysis included: red blood cells (RBCs), hemoglobin (HGB), hematocrit (HCT), total leukocytes, granulocytes, lymphocytes, and monocyte count. The analysis was performed using a Mythic TM 80 Vet hematology analyzer (Plan-les-Ouates, Switzerland). Biochemical parameters, glucose and alanine aminotransferase (ALT), were measured using a Vetscan^®^ chemical analyzer (Abaxis/Zoetis, Parsippany, NJ, USA).

### 2.5. Statistical Analysis

Animals were randomly assigned to treatments using the randomization function in Excel (Microsoft Excel, Office 2019, Microsoft Corporation, 2026). Statistical analyses were performed using GraphPad Prism version 11.0.0 (GraphPad Inc., La Jolla, CA, USA). Body weight was analyzed by repeated measures ANOVA followed by Tukey’s multiple comparisons. All other data were analyzed using one- or two-way ANOVA followed by Tukey’s or Dunnet’s post hoc analyses for multiple comparisons across the groups. Statistical significance was set at *p* < 0.05.

## 3. Results

### 3.1. Body Weight Developed Normally Across EE Conditions Evaluated

**Figure 1:**
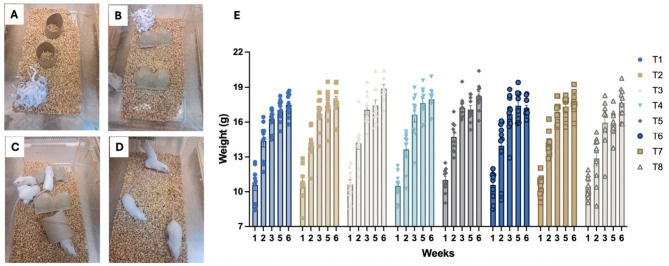
Body weight increased progressively over the six-week study period in all groups, irrespective of environmental enrichment conditions. (**A**–**D**) Representative images of selected treatment groups: T5 (**A**), T6 (**B**), T7 (**C**), and T8 (**D**). (**E**) Body weight changes for group over the six-week study period. Data are presented as mean ± SEM (*n* = 10 per group). The week-4 body weight data were excluded because of a technical error during data collection.

The animals were housed for six weeks under conditions that included different environmental enrichment materials or no enrichment (Figure 1A–D). Body weight was monitored weekly as an indicator of physiological adaptation and a potential stress response. As expected, all groups showed a progressive increase in body weight during the experimental period (Figure 1E, raw data Table S1), which is consistent with normal growth patterns for this strain. No significant differences were detected between groups at any time point.

### 3.2. Different EE Conditions Do Not Affect Locomotion but Do, in Some Cases, Promote Anxiolytic-like Behavior

**Figure 2:**
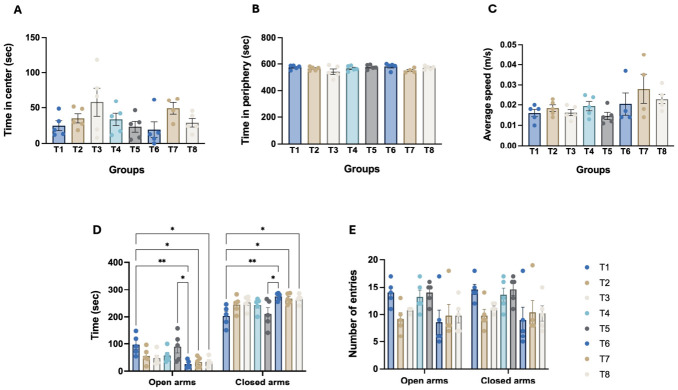
Groups T1 and T5 exhibited anxiolytic-like behavior. Locomotor and exploratory activities (**A**–**C**), as well as anxiety-like behavior (**D**,**E**), were evaluated at the end of the experimental period. Graphs represent time spent in the center (**A**), time spent in the periphery (**B**), and average speed (**C**) measured in the Open Field. In the Elevated Plus Maze, time spent (**D**) and number of entries (**E**) into the open and closed arms were analyzed. Data are presented as mean ± SEM (*n* = 5 per group). * *p* < 0.05; ** *p* < 0.01.

To determine whether different environmental enrichment materials affected locomotor and exploratory behavior, animals were evaluated using the OF. No significant differences were observed between treatments in the time spent in the central zone (F (7,31) = 1.622, *p* = 0.1633) or the peripheral zone (F (7,31) = 1.622, *p* = 0.1663) or the average speed (F (7,32) = 1.600, *p* = 0.1712) (Figure 2A–C, raw data Table S2). The average speed remained within the expected physiological range (0.01–0.03 m/s), indicating no alterations in basal locomotor activity. Overall, these results suggest that the environmental enrichment did not significantly affect the general exploratory behavior or locomotor activity in the Open Field Test.

To assess anxiety-like behavior, we used the EPM. Mice spent significantly more time in the closed arms than in the open arms (F (1,64) = 829.8, *p* < 0.0001), reflecting a typical anxiety-like response in this test. However, a significant interaction was observed (F (7,64) = 7.786, *p* < 0.0001). Post hoc analyses revealed mice in T1 and T5 spent significantly less time in the closed arms and more time in the open arms compared to groups T6, T7, and/or T8, indicating a reduction in anxiety-like behavior (Figure 2D, raw data Table S3). No significant differences were observed between groups in the number of entries into the open arms (Figure 2E, raw data Table S3), suggesting similar levels of overall exploratory activity between the groups.

### 3.3. White Blood Cells Were Induced by the Combination of Cardboard Rolls and Maple Cardboard (T7)

Blood samples were collected for hematological analysis. No significant differences were observed in red blood cell parameters, including the RBC count, hemoglobin, and hematocrit (Table 1, raw data Table S4). The values obtained for the three analyzed parameters in all experimental groups were within the expected range for this mouse strain [18].

**Figure 3:**
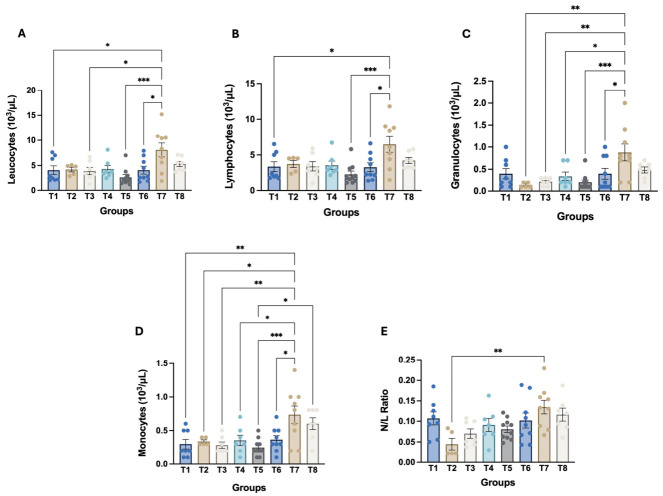
The combination of cardboard rolls and maple cardboard induced higher white blood cell counts. After 6 weeks of exposure to different enrichment materials, blood was collected, and the numbers of leukocytes (**A**), lymphocytes (**B**), granulocytes (**C**) and monocytes (**D**) were analyzed. (**E**) The ratio N/L was calculated. Data are represented as mean ± SEM (*n* = 7–8 per group). * *p* < 0.05; ** *p* < 0.01; *** *p* < 0.001.

Regarding white blood cell counts, no significant differences were generally observed between the enrichment groups and the control group (T8) (Figure 3A–D, raw data Table S5). However, significant differences were observed among EE groups for all leucocytes analyzed. Leucocyte counts showed a significant overall effect of the group (F (7,54) = 4.006, *p* = 0.0013), with the post hoc analysis indicating higher values in T7 compared to T1, T3, T5, and T6 (Figure 3A). Similarly, lymphocyte counts differed significantly among groups (F (7,54) = 3.552, *p* = 0.0033), with T7 showing differences relative to T1, T5, and T6 (Figure 3B). Additionally, granulocyte counts also showed a significant group effect (F (7,54) = 4.472, *p* = 0.0006), with higher values in T7 compared to T2–T6 and a trend toward increases versus T1 and T8 (*p* = 0.0543 and *p* = 0.0841, respectively) (Figure 3C). For monocyte counts, a significant group effect was observed (F (7,54) = 5.082, *p* = 0.0002), with T7 differing from groups T1–T6 (Figure 3D). We also observed a statistically significant difference between T5 and T8 (*p* = 0.0404) and trends for T1 and T3 relative to T8 (*p* = 0.0564 and 0.0515, respectively).

The ratio between neutrophils and lymphocytes (N/L) was explored as a biomarker for stress [19] (Figure 3E). The N/L ratio was significantly higher in T7 compared to T2 and a trend was observed between T7 and T3 (*p* = 0.0617) and between T8 and T2 (*p* = 0.0878). Collectively, these results suggest that the combination of cardboard rolls and maple cardboard (T7) shows the strongest effect on white blood cell counts.

### 3.4. Alanine Aminotransferase (ALT) Enzyme Levels Were Affected by Mouse Exposure to Cardboard Rolls and Maple Cardboard

**Figure 4:**
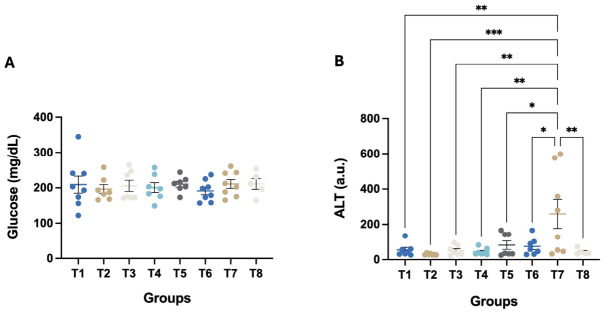
The T7 condition affected levels of ALT in female BALB/c mice. Concentrations of glucose (**A**) (*n* = 8) and ALT (**B**) (*n* = 7–8) were measured in the blood at the end of the experimental period. Data are represented as mean ± SEM. * *p* < 0.05; ** *p* < 0.01; *** *p* < 0.001.

To assess the potential effects of environmental enrichment on metabolic and hepatic parameters, glucose and ALT levels were measured at the end of experimental period (raw data Table S6). Glucose concentrations did not differ significantly among the experimental groups (F (7,49) = 0.2562, *p* = 0.9677) (Figure 4A). In contrast, ALT levels differed significantly among groups (F (7,47) = 4.582, *p* = 0.0006), and the post hoc analysis revealed that mice in the T7 group exhibited significantly higher ALT concentrations than those in the remaining groups (Figure 4B). Importantly, ALT values in T7 exceeded the reference range reported for this mouse strain [20]. Overall, these findings indicate that the enrichment strategies evaluated did not substantially affect glucose homeostasis, whereas the T7 condition was associated with elevated ALT concentrations.

## 4. Discussion

Environmental enrichment (EE) is widely recognized as a key refinement strategy to improve the welfare of laboratory animals by promoting the expression of species-specific behaviors and improving the animals’ ability to cope with environmental challenges [2,21,22,23,24]. In the present study, we investigated the effects of different environmental enrichment materials, used either alone or in combinations, on the welfare of female BALB/c mice housed under standard laboratory conditions. Welfare was evaluated through behavioral assessments and the analysis of physiological and biochemical parameters [25]. Our results reveal that the type and combination of enrichment materials influence several welfare-related outcomes. This study contributes to current efforts to promote and ensure the welfare of rodents maintained in laboratory settings.

The type of environmental enrichment has been shown to influence mouse behavior in a strain- and sex-dependent manner [26]. In male CBA/2J mice, aggressive behavior has been observed under enriched housing conditions and has been associated with unstable dominance hierarchies and social status-dependent neuroendocrine alterations [27]. In addition, elevated testosterone levels have been reported in males of several strains, including BALB/c, exposed to environmental enrichment, suggesting a potential relationship between aggression, testosterone levels, and enrichment conditions [28]. To minimize the potential confounding effects of aggression, we evaluated the effects of different environmental enrichment materials in female mice.

The selection of enrichment materials should be guided by the animals’ perspective and how they interact with the available resources [29]. Kraft paper was consistently used by the mice for nest building, a highly motivated species-specific behavior that contributes to thermoregulation and the maintenance of a favorable cage microenvironment [30]. While the maple cardboard was extensively manipulated and chewed, cardboard rolls served primarily as shelters, indicating their distinct functional roles. Although no formal preference test was performed, all materials were actively used by the animals. Direct observation throughout the study revealed no stereotypic or agonistic behaviors in any group.

Body weight is a fundamental indicator of overall health and physiological adaptation in laboratory animals. The enrichment strategies evaluated in this study do not appear to adversely affect growth performance, since the body weight increased progressively in all groups throughout the experimental period, and no significant differences were observed among the treatment groups. Our results are consistent with previous studies that reported minimal or no impact on the body weight of animals housed in the presence or absence of environmental enrichment [31]. Other studies, however, have shown changes in body weight when animals were housed in enrichment conditions [32]. These apparent contradictions across studies may be explained by differences in animal strain; environmental conditions (e.g., housing density and macroenvironmental factors); and/or the type, duration, and implementation of the enrichment strategies used.

A limitation of this study is that the cage temperature was not measured individually for each housing cage. Previous studies have reported that mice housed at ambient temperatures of 20–22 °C may experience mild chronic cold stress [33]. Although the mean cage temperature in our facility was below the recommended range [34], we observed no alterations in species-specific behaviors or body weight gain over time in any of the experimental groups. We believe that the social enrichment provided by the group housing may have enabled the animals to maintain thermoneutrality through social thermoregulation [35], which may explain why no evidence suggestive of cold stress was observed, even in mice housed without additional environmental enrichment or without Kraft paper as a nesting material.

Baseline locomotor activity and anxiety behaviors were evaluated using the Open Field (OF) and Elevated Plus Maze (EPM) tests, since previous studies have reported context-dependent effects of environmental enrichment on anxiety-related behaviors. In the present study, the enrichment used did not affect general locomotor activity and exploratory behavior. These findings indicate that the observed behavioral results were not associated with changes in basal activity levels, consistent with previous reports [36,37,38].

In contrast, the EPM revealed that specific enrichment combinations influenced anxiety-like behavior. Mice exposed to all three enrichment materials (T1) or to cardboard rolls plus Kraft paper (T5) showed profiles consistent with reduced anxiety-like behavior [39], which is associated with environmental adaptations and animal welfare [40]. These findings support the observation that the use of multiple materials promotes the etiological expression of species-specific behaviors, providing a greater welfare benefit than single-item enrichment. Interestingly, not all enrichment combinations produced positive behavioral outcomes. The groups exposed to maple cardboard and Kraft paper (T6) and cardboard rolls plus maple cardboard (T7) exhibited the lowest open arm exploration time, indicating an anxiogenic effect of these combinations. Thus, the effects of enrichment are not necessarily additive, and specific combinations of materials may differ in their functional value to mice. The behavioral benefit observed in T1 may result from the simultaneous availability of nesting material and manipulable objects. Moreover, the effects of environmental enrichment materials on parameters associated with anxiety are not uniform and depend on multiple experimental factors, including the type of enrichment, duration, strain, sex, and behavioral test used. As expected, the absence of environmental enrichment (T8 group) was also associated with a reduced exploration of the open arms, suggesting an anxiogenic-like behavioral profile in this group.

A potential limitation of this study is that the estrous cycle was not assessed before the OF and EPM tests. Although several studies have reported that the estrous cycle can influence anxiety-related behaviors [41,42,43], others have found little or no effect on these behavioral outcomes [44,45,46,47]. These discrepancies may reflect differences in the mouse strain, age, housing conditions, or experimental protocols. Notably, recent studies using machine learning approaches have shown that exploratory behavior in the OF is more variable in males than in females [48], suggesting that the contribution of the estrous cycle to anxiety-like and exploratory behaviors may be smaller than previously assumed. While we cannot rule out a potential influence of the estrous cycle on the behavioral outcomes observed in this study, we found no differences in the OF performance among the treatment groups that could be attributed to estrous stages. In the EPM, we observed an increase in the time spent in the open arms, suggesting an anxiolytic effect that may be associated with exposure to the environmental enrichment materials. Within the present experimental design, we cannot rule out the possibility that fluctuations in the estrous cycle contributed to this effect. Future studies should investigate this potential contribution. Nevertheless, we interpret our findings as indicative of a positive effect of EE on animal well-being, regardless of whether this effect is influenced by the estrous cycle.

The assessment of animal welfare should not be based exclusively on behavioral parameters but should be complemented by physiological indicators that allow for the identification of potential alterations in the state of welfare. In this context, the evaluation of hematological and biochemical parameters takes on special relevance. Reports indicate that recurrent exposure to a stressor is associated with cell mobilization and increased myelopoiesis in the bone marrow, followed by high levels of neutrophils and monocytes in the blood [49]. In the present study, mice exposed to cardboard rolls and maple cardboard (T7) exhibited the highest circulating granulocyte, leucocyte, lymphocyte and monocyte counts, whereas animals housed with cardboard rolls and Kraft paper (T5) showed the lowest values for these hematological variables. These findings suggest that different enrichment combinations may differentially influence immune-related parameters. For example, the T7 enrichment condition may be associated with increased immune cell mobilization or immune surveillance activity [50]. However, further studies are required to determine whether these responses reflect enhanced immune competence, increased physiological activation, or other underlying mechanisms.

The enhanced immune activity observed in the T7 group occurred in the absence of beneficial effects on anxiety-like behaviors, suggesting that these materials may have promoted immune activation without producing a measurable improvement in animal welfare. Notably, the combination of cardboard rolls and Kraft paper (T5) was associated with lower levels of inflammatory or immune activation states, with cell counts within the reported normal range for this strain [51]. This profile may be related to the reduced anxiety-like phenotype observed in the EPM. Similarly, the combination of cardboard rolls, Kraft paper and maple cardboard (T1) resulted in moderate leucocyte, lymphocyte, monocyte, and granulocyte counts, suggesting normal immune activity along with anxiolytic-like effects in the EPM. Collectively, these findings suggest that enrichment conditions including cardboard rolls and Kraft paper (T5), either alone or combined with maple cardboard (T1), may be associated with lower chronic stress levels and improved welfare outcomes.

The involvement of the immune system in stress-related disorders has been increasingly documented in both mice and humans [52,53]. In this context, variations in circulating monocyte levels have been proposed as a relevant indicator of stress responses. Previous studies have reported both decreases and increases [54] in monocyte counts following chronic or acute stress exposure. In this study, monocyte counts were highest in T7 and in the non-enriched group (T8), whereas T5 showed significantly lower counts, with the remaining groups exhibiting intermediate values. These findings suggest that most enrichment strategies may have contributed to limiting monocyte accumulation compared with T7 and the non-enriched condition. When considered together with the behavioral and hematological results, the enrichment conditions of T5 and T1 appear to provide the most favorable balance between reduced anxiety-like behavior and the absence of evidence for increased immune activation.

Elevated granulocyte counts (neutrophilia) and reduced lymphocyte counts (lymphopenia) have been recognized as hematological indicators associated with chronic stress in mice [55]. Consequently, an increased neutrophil-to-lymphocyte (N/L) ratio has been closely associated with stress, inflammation, and anxiety-related behaviors and has been validated as a reliable biomarker of stress in murine models [56,57]. The higher N/L ratio observed in the T7 group may reflect physiological stress associated with the enrichment condition. Interestingly, the N/L ratio in T7 was comparable to that observed in the non-enriched group (T8), further supporting the possibility that this housing condition may induce a stress-related physiological response. On the contrary, animals exposed to maple cardboard alone (T2) and cardboard rolls alone (T3) tended toward lower N/L ratios, despite showing no significant modification of anxiety phenotypes. This finding highlights that environmental enrichment can alter physiological indicators of stress independently of detectable behavioral changes. Collectively, these results underscore the importance of adopting a multidimensional approach to welfare assessment, as animal welfare is a complex and multifactorial phenomenon that cannot be reliably evaluated using a single parameter.

Measures of animal health are often determined by blood biochemical parameters. One metabolic parameter that can be considered an indicator of stress or malaise in laboratory mice is glucose [58]. According to this criterion, approximately 40% of stressed mice exhibited a high glucose phenotype (glucose > 150 mg/dL), a measure associated with greater stress susceptibility. Our study reports normal glucose levels for all groups evaluated, suggesting that the EE materials used did not alter the baseline metabolic rate. Importantly, these findings indicate that the enrichment materials are safe for use in mice, as they do not appear to be toxic or adversely affect metabolism, even when chewed during normal exploratory behavior.

Altered ALT levels may indicate liver damage attributable to hepatocellular stress [59]. We observed a significant increase in levels of the ALT enzyme in the T7 group compared to all other groups, which showed normal levels of circulating ALT. Although the chemical composition of the environmental enrichment materials was not characterized, these results support the conclusion that the materials were not toxic to the animals. Collectively, the ALT data combined with white blood cell data suggest that the animals exposed to cardboard rolls and maple cardboard (T7) likely had systemic inflammation, which may be related to stress. Despite the SPF housing conditions, with continuous sentinel monitoring, we cannot formally rule out an infectious cause for this phenotype. Nevertheless, the normal growth patterns and species-specific behavior observed in this group do not support the presence of an underlying disease condition.

## 5. Conclusions

Taken together, our results suggest that enrichment strategies incorporating multiple complementary materials are more effective than single-material approaches in promoting positive behavioral outcomes. Among the treatments tested, the combination of maple cardboard, cardboard rolls, and Kraft paper (T1) produced the most favorable overall behavioral profile, while the combination of cardboard rolls and Kraft paper (T5) also showed substantial benefits and was associated with relatively low immune activation. In contrast, cardboard rolls and maple cardboard (T7) produced pronounced hematological responses without comparable behavioral improvements, indicating that physiological stimulation and behavioral welfare may not always occur in parallel.

Overall, the enrichment materials evaluated are compatible with the housing conditions in the animal facilities, as they did not introduce significant variability in most of the welfare indicators assessed. However, the significant differences observed in white blood cell counts (monocytes, lymphocytes, and granulocytes), the neutrophil to lymphocyte ratio, and the assessment of behavior in the Elevated Plus Maze suggest that when cardboard rolls and Kraft papers are used and combined with maple cardboard, animals experience improvements in welfare.

## Figures and Tables

**Table 1:** Red blood cell count (RBC), hemoglobin concentration (HGB), and hematocrit (HCT) values in the different experimental groups.

Group	RBC (Cells × 10^6^/mL)	HCT (%)	HGB (g/dL)
T1	8.37 ± 1.20	40.45 ± 5.73	14.23 ± 2.04
T2	9.04 ± 0.48	41.74 ± 1.59	15.60 ± 0.89
T3	7.92 ± 1.72	37.94 ± 8.10	13.14 ± 3.44
T4	8.98 ± 0.56	43.07 ± 2.67	15.01 ± 1.18
T5	7.15 ± 2.67	34.35 ± 11.98	12.06 ± 4.57
T6	8.91 ± 0.62	42.22 ± 3.31	15.03 ± 1.18
T7	8.92 ± 0.62	42.81 ± 3.19	15.17 ± 1.36
T8	8.72 ± 1.82	42.71 ± 8.14	14.80 ± 3.75
Group differences	F (7,54) = 1.857, *p* = 0.0951	F (7,54) = 1.854, *p* = 0.0956	F (7,54) = 1.650, *p* = 0.1413

The data are expressed as the mean value ± SD.

## Data Availability

The original contributions presented in this study are included in the Supplementary Materials. Further inquiries can be directed to the corresponding authors.

## Supplementary Materials

The following supporting information can be downloaded at https://www.mdpi.com/article/10.3390/ani16142211/s1.

## Abbreviations

The following abbreviations are used in this manuscript:

EE	Enrichment Environmental
ARRIVE	Animal Research: Reporting of In Vivo Experiments
F18	Generation 18
T1 to T8	Treatment 1 to Treatment 8
OF	Open Field
EPM	Elevated Plus Maze
RBC	Red Blood Cell
HCT	Hematocrit
HGB	Hemoglobin
ALT	Alanine Amine Transferase
N/L	Neutrophil/Lymphocyte
IACUC	Institutional Animal Care and Use Committee
INDICASAT	Institute of Investigation and Service High Technology
